# A Systematic Review on Serious Games in Attention Rehabilitation and Their Effects

**DOI:** 10.1155/2022/2017975

**Published:** 2022-02-26

**Authors:** Leila Shahmoradi, Fatemeh Mohammadian, Meysam Rahmani Katigari

**Affiliations:** ^1^Department of Health Information Management, School of Allied Medical Sciences, Tehran University of Medical Sciences, Tehran 1417744361, Iran; ^2^Department of Psychiatry, Roozbeh Hospital, Tehran University of Medical Sciences, Tehran 13337159140, Iran

## Abstract

Attention is a basic and main mental task and can play an important role in the functioning of other brain abilities such as intelligence, memory, learning, and perception, and its deficit occurs in 80% of patients with traumatic brain injury. The use of game-based tools for rehabilitation is rapidly expanding. Cognitive rehabilitation via video games is an emerging hot topic in cognitive science. Serious games serve a specific purpose in addition to entertainment. They can be more engaging than exercises since they replace reward and motivation systems with real-world motivations as a complement for rehabilitation activities. This study was aimed at identifying and categorizing serious computer games used for attention rehabilitation and evaluating their effects. Six electronic databases (Scopus, PubMed, ISI, Embase, IEEE, and Cochrane) were searched in August 2021. The search strategy consisted of three main concepts of “serious game”, “cognitive deficits”, and “cognitive rehabilitation”. The inclusion criteria were (1) journal articles, (2) English language, (3) being published in the last 10 years, (4) human participants, and (5) game-based intervention. In the 30 included studies, 22 unique games were utilized for attention rehabilitation. Lumosity (20%), Brain Age (Dr. Kawashima's Brain Training) (10%), and MoHRS (6.66%) were the most common games among the studies. There were (57%) casual, (23%) action, (10%) simulation, and (10%) multiple genres. Of the 47 tools used in the studies, 5 utilized cross-modal oddball attention tasks, 4 utilized game performance, 3 utilized the paced auditory serial additional test (PASAT), and the rest employed other tools. A total of 73 outcome measures were related to attention, 42 measures did not have significant results, 30 were significantly improved, 1 was significantly deteriorated, and 4 articles did not have any specific measures for attention evaluation. Thus, the results revealed the positive effect of serious games on attention. However, issues such as absence of scientific teams, the variety of the disorders that cause defects, the variety of criteria, differences in measurements, lack of long-term follow-up, insufficient RCT studies, and small sample sizes should be considered when designing, developing, and using game-based systems to prevent bias.

## 1. Introduction

Attention is defined as a set of complex psychological functions that include focusing or engaging with the goal, enduring, and being alert for a long time. It is a feature in the human brain that allows a limited amount of information to be actively processed. This information is taken from the vast amount of information available to the senses, stored memory, and other cognitive processes [1]. It is the basis of cognitive functions because any defect in its function can decrease cognitive efficiency [2], and its improvement can greatly contribute to the rehabilitation process of stroke patients [3]. Attention is a major mental task and can also play an important role in the functioning of other cerebral abilities such as intelligence, memory, and perception [4]. Attention deficit can be caused by various diseases and disorders such as multiple sclerosis, traumatic brain injuries, and stroke. In each of these neuropsychological situations, there are different physical and cognitive symptoms in patients [5].

The attention control system is one of the most complex control procedures in our nervous system. Different networks distributed in the brain are involved in different aspects of the attention control system. Interactions between and within these networks of attention are mediated by various inhibitory and excitatory neurotransmitters. These neurotransmitters play a vital role in the proper functioning of the attention control system, and their interaction is essential. Any defect in neurotransmitter interactions can lead to dysfunction of attention networks and consequently dysfunction of the attention control system [6].

Attention is defined as awareness of what is happening around us [7]. Its deficit is very common and usually occurs in 80% of patients with traumatic brain injury, slowing down the patient's reactions in daily life and increasing irritability [8]. The model of Sohlberg and Mateer [9] classifies attention into five different categories of focused, sustained, selective, alternating, and divided attention [9]. In terms of types of stimuli and due to the existence of five senses in the human body, it is divided into 5 categories: visual, auditory, tactile, olfactory, and gustatory [6]. Also, attention can be divided into two broad categories: intensive processes, such as alertness and vigilance, and selective attention processes, such as focused and divided attention. Aspects of intensity could be a prerequisite for more complex aspects, such as selective [10, 11]. In fact, lack of attention is associated with problems with balance, daily life functions, and falls [12]. Due to the high impact of attention deficit on other cognitive and physical functions, many researchers have tried to provide effective treatment options for this deficit [13].

Researchers are now well aware that the brain is a more flexible organ than previously thought and is able to significantly repair damage by reorganizing itself, which is the basis of functional rehabilitation. This feature is called neuroplasticity of the brain [14]. It is shown that the brain can repair itself after injury through repetitive, intensive, and task-oriented exercises [15]. Cognitive rehabilitation, behaviour adjustment, psychological management, education, and individual and family counselling are the primary methods of treatment in the rehabilitation of attention deficits [16]. Many researchers have emphasized the importance of cognitive rehabilitation in reducing behavioural and cognitive consequences and promoting independence and quality of life [14, 17, 18]. Cognitive rehabilitation currently generally uses one of the two approaches to treating cognitive deficits. (a) The therapeutic or restitution approach seeks to directly retrain impaired cognitive function. The basic rationale for this approach is the notion that practicing carefully selected exercises improves damaged neural circuits and restores function in the damaged attention processes themselves. (b) The alternative or compensation approach helps people with attention deficits learn or relearn how to perform specific skills. The basic rationale for this approach is that new neuropsychological processes replace the damaged areas of the brain through practice and develop individual skills [19, 20]. Most studies have pointed to the positive effect of cognitive rehabilitation on attention deficit due to various diseases in both approaches, especially in the compensation approach [21–23], but at the same time, some studies are ambiguous about this positive effect in the long time [23, 24].

Still, the most important challenge for therapists is how to encourage patients to perform rehabilitation programs frequently [25, 26]. Today, cognitive rehabilitation methods can be divided into two main categories: traditional methods and computer-based methods. Traditional methods use noncomputer neuropsychological techniques to improve attention and concentration deficits. The traditional rehabilitation is done face-to-face; that is, one or sometimes several therapists work with a patient [27]. These methods have many limitations such as lack of access in all places, high travel costs, lack of accurate monitoring, dearth of information about the patient's performance, and being dull due to their repetitive nature [28, 29]. Computer-based methods use similar neuropsychological processes but utilize the computer for training as an adjunct to face-to-face rehabilitation that will solve the problems associated with traditional methods [29–31]. Several studies suggest that providing feedback, training strategies, and intervention control by the therapist, along with technology, can improve outcomes [31–33].

Over the past few decades, evidence has highlighted the positive impact of computer-based rehabilitation programs on a variety of deficiencies [31, 34–38]. Among these interventions, the use of game-based tools for rehabilitation is rapidly increasing [39]. Serious games are games that serve a specific purpose in addition to entertainment [40]. The use of serious games in physical rehabilitation has a long history, but this method in cognitive rehabilitation, although scientifically a hot topic, is still not practically and widely practiced [41, 42]. Serious games can be more engaging than exercises because they replace reward and motivation systems with real-world motivations as a complement to rehabilitation activities. People can be immersed in the game world, and their ability and knowledge can be improved without any danger [43]. Other features of these games are increasing the quality and effectiveness of rehabilitation, providing different levels of play to people according to the severity of defects and overcoming the limited resources and facilities of conventional rehabilitation methods [44]. Coles et al. [45] used computer games to teach safety knowledge to children with cognitive impairments, and Prang et al. [46] utilized computer games to rehabilitate disabled patients. Traditional rehabilitation exercises require repetitive and dull activities that are not completed by patients. Moreover, most people today are familiar with digital environments, especially mobile phones. Therefore, health professionals are interested in using computer games for rehabilitation purposes, which will both enhance motivation and determine the outcome of rehabilitation [47, 48].

In this qualitative literature review, we summarize findings about serious games in attention rehabilitation. The objectives of this review are as follows:

1. To investigate and identify the existing literature on the application of serious games for attention rehabilitation
2. To map and categorize the literature according to the study purpose, target group, measures methods, features and capabilities of games, analytical results, and interpretation
3. To evaluate the effects of serious games in cognitive rehabilitation

## 2. Materials and Methods

Herein, we present a systematic literature review of serious games for attention rehabilitation based on the PRISMA checklist 2020 [49]. This review identified and classified relevant studies on serious games and summarized the findings and gaps. To be flexible in the search, we used parallel and iterative processes for screening, classification, and review phases.

### 2.1. Eligibility Criteria

The eligibility criteria were as follows: (1) being a journal article, (2) being written in English, (3) being published in the last 10 years, (4) human participants, and (5) providing any game-based intervention. Studies would be excluded from the study if they were (1) unrelated and (2) duplicated; (3) had unavailable full texts or were abstract-only studies; (4) were of other types, e.g., reviews, (5) involved no video games or cognitive rehabilitation; and (6) were protocols and studies only about Exergames.

### 2.2. Information Sources and Search Strategy

According to the AMSTAR guidelines, at least two databases have to be searched in a systematic review [50]. To obtain more accurate and exhaustive results, we increased the number of searched databases. Six electronic databases (Scopus, PubMed, ISI, Embase, IEEE, and Cochrane) were searched on 2 August 2021 for articles published from January 2011 to August 2021. In addition, to ensure the search of all related articles and reduce the possibility of bias, a manual search was performed using two methods: (1) checking the references of the related papers and (2) using the Google Scholar search engine. We adopted the PICO approach to prepare the search terms [51]. The research team jointly selected three different categories of keywords “serious game”, “cognitive deficits”, and “cognitive rehabilitation” to achieve the objectives of the study and the exhaustiveness and sensitivity of the search. Then, we used MeSH, Emtree, and other related papers to find all the keywords related to these categories. During the primary search, we found that some articles with topics and themes cognitive rehabilitation, executive function, memory, perception, problem solving, etc. also considered attention along with these topics; thus, to cover these articles as well, we used the term cognitive as the more general term instead of attention. First, a standard search was performed in PubMed; then, in other databases, this strategy was modified according to the specific symbols and search methods in that database to obtain the most relevant related results. Our search strategy was a combination of words such as computer game^∗^ OR video game^∗^ OR online game^∗^ OR applied game^∗^ OR serious game^∗^ OR gamification OR virtual game OR Mobile game^∗^ AND Cognitive Dysfunction OR Cognit^∗^ OR Attention defect^∗^ OR attention^∗^ OR perception OR concentration AND Remediat^∗^ OR Rehabilitat^∗^ OR Train^∗^ OR Therap^∗^ OR Readapt^∗^. The search strategies for each database are listed in supplementary 1 Tables S1–S3.

### 2.3. Selection and Data Collection Process

After importing the citations to EndNote 20, duplicates were detected and deleted by this software. To select studies, they were read in two phases, including their title or abstract and a full-text review by two independent reviewers. Disagreements in selection were resolved by a third reviewer. Studies were eligible for data extraction if they met all the inclusion criteria and did not meet the exclusion criteria in the opinion of the reviewers. Then, a Microsoft Excel 2016 data extraction spreadsheet was designed and used by all team members to collect data. The spreadsheet items included the following: title, journal, author, year, volume, pages, abstract, keywords, objective, disease, country, setting, target group, mean age of participants, name of the game, platform, description of the game, game genre, sample size, intervention group, control group, evaluation methods, outcome measures, results, and statistical methods. In the spreadsheet, some responses were in the open-answer format and some in the closed-answer format. The reviewers could choose the not clear option if they could not find the answer. To extract more information about games that were not fully described by the included article, Internet resources and other articles were searched. We also searched the websites of the games to obtain the latest release information.

### 2.4. Risk of Bias Assessment

Because we aimed to provide a classification of articles and games available for attention rehabilitation and evaluate their overall effects, at this phase, we did not intend to conduct a meta-analysis on the data; therefore, we did not perform a quality assessment of the included studies.

### 2.5. Synthesis Methods and Analysis

According to the objectives, comparative tables and figures are presented to describe and categorize the results. With a qualitative analysis method, we combined and analysed the results and no meta-analysis was performed due to the heterogeneity of the outcome measures and differences in the populations. Prior to this step, data were checked and refined to provide better results. Excel 2016 was used to present and analyse the data.

## 3. Results

### 3.1. Selection of Studies

**Figure 1:**
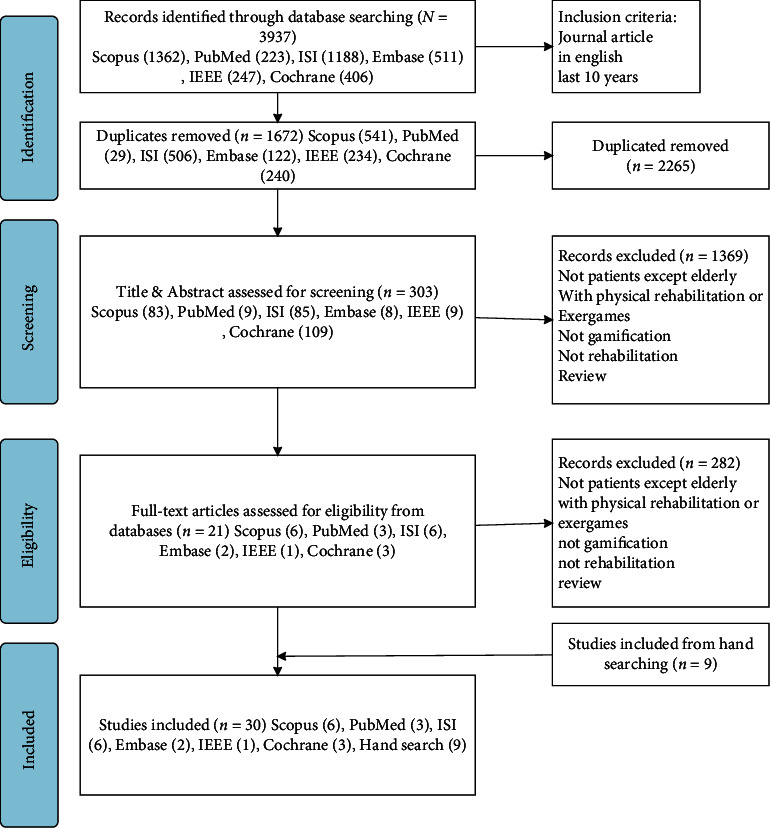
PRISMA flow diagram of the study selection process.

The Preferred Reporting Items for Systematic Reviews and Meta-Analyses (PRISMA) flow diagram of the study selection process is displayed in Figure 1. Initially, 3,937 studies were obtained from six scientific databases; after removing the duplicates, the remaining 1,672 articles were screened based on the titles and abstracts. The screening process resulted in the exclusion of 1,369 articles based on the inclusion and exclusion criteria. Then, 303 articles were downloaded in full text for further screening. Of these, 282 were excluded after reviewing their full text for various reasons, e.g., administering the intervention to nonpatients, games with physical activities, lack of gamification, noncognitive rehabilitation intervention, or being review articles. From these databases, 21 articles entered the final phase of inclusion, and 9 articles were manually searched. Finally, 30 articles were selected for data extraction. The results are visualized using the PRISMA flow diagram in Figure 1.

### 3.2. Study Characteristics

**Figure 2:**
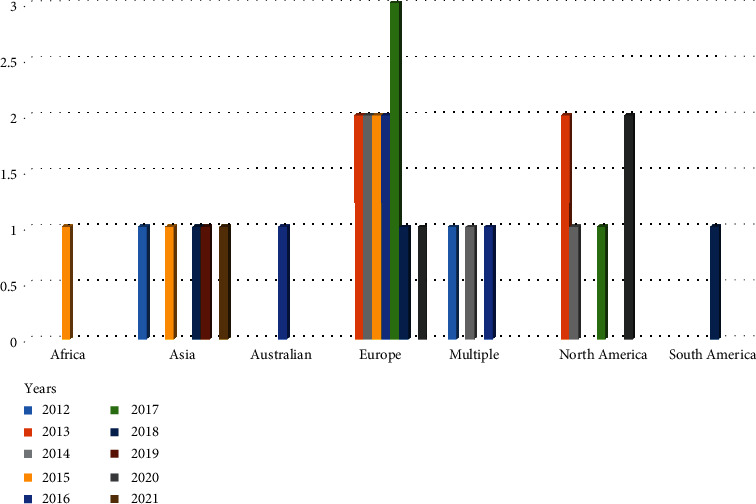
Tendency of studies based on 10-year periods worldwide.

**Figure 3:**
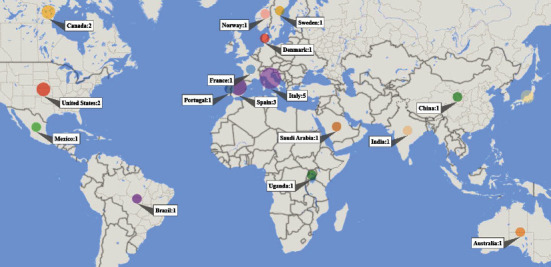
The distribution of papers by their conducted countries.

Of the 30 articles included in the review, all were published in English from 2012 to 2021, with most articles being published during 2013-2017 (four per year). The journals with the highest numbers of publications were *Frontiers in Aging Neuroscience* (*n* = 3) and *PLOS One* (*n* = 2), and 13 of 30 of these journals (43%) are ranked in quartile 1. All the other journals had published only one included article. Most of the studies were conducted in Europe 13 (43%), followed by 6 (20%) in North America, 5 (17%) in Asia, 3 (10%) in multiple countries, and other in Australia (1, 3%), Africa (1, 3%), and South America (1, 3%) (Figures 2 and 3).

The mean age of the participants ranged from 5.2 to 79.4 years (Table 1). The highest target group of the games was the elderly 10 (33%) and those with traumatic brain injury 6 (20%). Other target diseases include autism spectrum disorder, ADHD, at-risk children, developmental disabilities, HIV, dementia, dyslexia, neglect disorder, neurocognitive disorders, stroke, and multiple sclerosis (MS). Evidently, the target groups in the studies were very diverse. Moreover, 12 (40%) of the studies were RCTs, while the rest were of other types such as prepost (6, 20%), pilot RCT (4, 13%), quasi-experimental (3, 10%), case series (2, 6.66%), single-subject (2, 6.66%), or uncontrolled clinical trial (1, 3.33%). Most studies were conducted in Italy (4, 13%) and Spain (4, 13%), and some studies were the result of the collaboration of several countries.

All included interventions belonged to different types of computer technologies involving gamification. On the other hand, there were various types of control groups. Most studies administered routine care to the control group (11 out of 30). However, one study compared a BCI video game (FarmerKeeper) with cartoons. Another study compared an active video game-based physical activity program with an aerobic exercise program. There was also a study comparing a popular brain training game (Brain Age) with a popular puzzle game (Tetris). One study also compared Lumosity and a simulation strategy game. In another study conducted using Lumosity for the intervention group, the control group participated in discussion sessions about general topics related to aging. A study compared Brainastic computerized cognitive training (CCT) with watching videos on history, art, literature, and science plus physical exercise as a control group. One study compared preselected games (Kinect Adventures and Kinect Sports) with a balance platform therapy (BPT) by the Biodex Medical Systems. Furthermore, one study compared the Medal of Honor: Rising Sun (MoHRS) with three control groups, including a placebo control arcade game (Tetris), a useful field of view (UFOV) training program, and routine care.

Most studies had a sample size of more than 10 (25 out of 30). Studies with a small sample were often comprised a virtual reality (VR) or augmented reality (AR) intervention group (8, 9, 29). The largest sample size was 232 and 157, respectively (24, 5), while the other sample sizes were below 60.

### 3.3. Mapping Serious Games in Attention Rehabilitation

In the included studies, 22 unique games were used for attention rehabilitation. Lumosity (6, 20%), Brain Age (Dr. Kawashima's Brain Training) (3, 10%), and MoHRS (2, 6.66%) were the most commonly used games. Moreover, 9 (40%) games were developed by the authors of the articles, 10 (45%) games by a commercial group, and 3 (13%) by other scientific groups. The included games had a variety of platforms. The PC application platform was the most frequent (9, 41%), followed by Kinect console (3, 14%), web application (3, 14%), augmented reality system (2, 9%), Nintendo console (2, 9%), PlayStation 2 (1, 5%), tablet application (1, 5%), and VR system (1, 5%).

**Figure 4:**
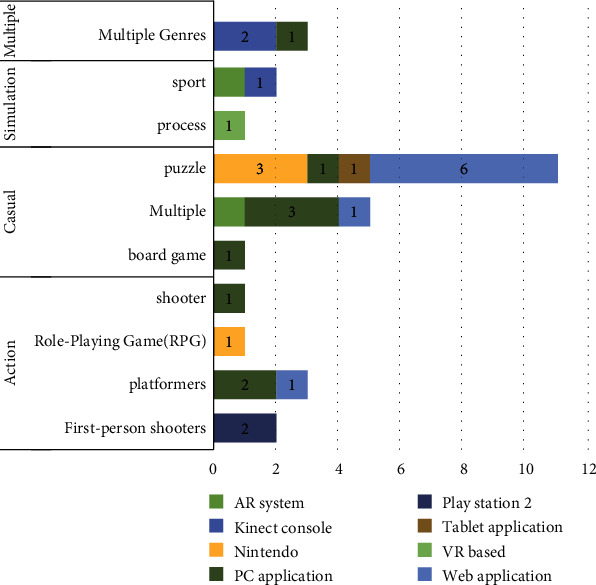
Genres and subgenres of games according to the platform. AR: augmented reality; VR: virtual reality.

Video game genre refers to categories of games with similar gameplay characteristics, usually not defined by the story and the setting (unlike cinema) but by how the player interacts with the game [81]. Genres cover a wide range of games and are further branched into subgenres. For example, a simulation game is classified into subgenres such as sports and process games [81]. Of all the included articles, there were 17 (57%) casual, 7 (23%) action, 3 (10%) simulation, and 3 (10%) multiple genres. Among the casual genre studies, there were 1 (6%) item with board games, 11 (65%) puzzles, and 5 (29%) multiple subgenres. In the action genre, there were 3 (43%) items with first-person shooters, 3 (43%) platformers, and 1 (14%) role-playing game (RPG) subgenre. Of the simulation genre studies, there were 1 (33%) item with process and 2 (67%) sport. All multiple genres also had multiple subgenres (3, 10%).  Figure 4 illustrates the genres and subgenres of games according to the platforms used in the articles.

### 3.4. Brief Description of the Games Involved

In this section, we will briefly describe the features of the games included in the study. All the games along with some of their features are listed in Table 2.

FarmerKeeper is a brain-computer interface video game developed by Mercado et al. to support neurofeedback training for children with autism spectrum disorder [52]. Neurofeedback can regenerate and retrain brain activity to enhance cognitive function in healthy individuals and those with certain developmental neurological conditions [82, 83]. The game takes place on a farm. The object is to keep children's attention above a certain threshold to control a runner who is looking for lost farm animals to bring them back to their pen.

Dr. Kawashima Brain Training for the Nintendo Switch is the fifth entry in the Brain Age puzzle video game series. It is based on research by neurologist Ryuta Kawashima whose avatar guides the player throughout the game. The Italian version of the game was used in the study by De Giglio et al. [53]. The minigames included in this game set include calculations, voice calculation, reading aloud, low to high, syllable count, head count, triangle math, and time lapse. In calculation, simple math questions appear on the screen very quickly and the player has to write the answer on the touch screen. In voice calculation, the answers must be said aloud. In reading aloud, a piece of a classic story should be read as soon as possible. In low to high, the position of numbers that appear on the screen for a short time should be memorized, and the player should demonstrate numbers from lowest to highest. In syllable count, the number of syllables in each sentence is counted. In head count, the screen above shows a group of people. After a few seconds, they are enclosed by a house and soon start coming in and out of the house. The player must count the number of people currently in the house. In triangle math, equations consist of 3 numbers and 2 operations and the player must perform operations in two modes, e.g., 7 + 5 + 4 and (7 + 5) + (5 + 4). In time lapse, the time difference between the 2 analog hours must be calculated.

Labyrinth includes a little man moving in the maze to reach the goal. The game character is controlled with a joystick by the gamer. The level of difficulty changes according to the task. The game consists of two tasks: diamond task and snake task. Each task has eight difficulty levels on a continuum from lower (level 1) to higher levels (level 8). The purpose of the game character depends on the nature of the current task. In DT, the man must collect diamonds that are randomly distributed in the play area. In ST, he must avoid being caught by a snake and reach the shelter house which appears in a random place.

Lumosity includes 50 games, 10 of which are specifically related to attention (Assist Ants, Feel the Beat, Skyrise, Eagle Eye, Playing Koi, Trouble Brewing, Train of Thought, Lost in Migration, and Star Search). In the included studies that have used the Lumosity package, different minigames have been utilized. Here, we provide a brief description of the games related to attention only. Assist Ants is designed to challenge your divided attention. You need to ensure the safety of each ant by helping it avoid collisions. Feel the Beat practices a sense of timing and rhythm. You have to rely on audio cues and use your sense of rhythm to match the beats in each experiment. In Skyrise, the goal is to work on your field of vision. In this game, several squares with numbers inside them appear. You have to memorize all the numbers and select them in ascending numerical order. The main goal of Eagle Eye is to improve your peripheral vision. You need to focus on the white circle in the middle and memorize the symbol or number that appears there. The goal of Playing Koi is to feed all the fish in the pond only once. In Trouble Brewing, you have to prepare a certain number of coffee orders in 2 minutes. In Train of Thought, you must guide each train to the matching station by switching the route keys. In Lost in Migration, the herd stays at one point and your task is to determine the path that the middle bird is facing. In Star Search, players must quickly find a unique object among a set of items that have the same shape, color, movement, and texture.

Captain's Log marketed by Brain Train Corporation is a comprehensive suite of computer cognitive learning games consisting of more than 2,000 computer-based exercises targeting 20 cognitive skills. Training modules are presented as games in which students' performance is recorded and points are earned based on performance accuracy.

Rayman Raving Rabbids includes 75 minigames and has two modes of play: story mode and score mode. In the story mode, the game follows 15 days of Rayman's imprisonment by the Rabbids. Every day, Rayman must do at least three trials. In the score mode, players can repeat previous trials to improve their scores.

Kinect Sports Ultimate Collection simulates 13 sports: basketball, soccer, American football, bowling, beach volleyball, table tennis, boxing, golf, tennis, skiing, darts, baseball, and track and field. To improve performance, songs and comments are used to help control and play the games.

AR-Therapist is a simulated augmented reality (AR) environment using a simple game. The game simply simulates two balls in 3D; one is the target ball and the other is not. The player must hit the ball at a specified time which, if correct, will add to the value of the correct shots. Otherwise, the value is added to the errors.

CogARC uses the AR interaction technique and manipulation of tangible physical objects (cubes) for cognitive screening and training. This game includes 6 minigames. Game mechanics include challenges, competition, feedback, and rewards. The gameplay structure offers two modes: free and linear. The player can play the minigames freely (free mode), in any order and at the desired levels, or play all the minigames in a predetermined order (linear mode). The minigames include Shape Match in which similar shapes should be matched, Color Match where you have to match the meaning of one word with another word's color, Sum Tower that uses numbers to create the desired sum, Building Blocks where you should find the answer to simple arithmetic calculations, Pattern Memory where you have to memorize and recreate a 3 × 3 matrix pattern of colored tiles, and Word Game where words related to a specific topic must be found.

Medal of Honor: Rising Sun (MoHRS) is a first-person shooter video game, the fifth in a series of Medal of Honor released by EA Game. Rising Sun is set in World War II during the Pacific War and has single and multiplayer capabilities.

The VR-based serious game application comprises several daily life activities such as buying several items, finding the way to the minimarket, finding a virtual character dressed in yellow, and recognizing outdoor advertisements, devised to train cognitive functions.

The Brain Powered Games (BPG) package includes Butterfly, iSpy, Stampede, Whacky Animal, and Gone Fishing. Butterfly is a simple game in which a butterfly flies on the screen and the player must use a mouse or other input devices to move the butterfly when it stops moving at a random location. iSpy is a memory game that works based on a common learning and memory pattern. The player is asked to watch a scene. After a brief display, the image is removed with a short delay and then represented, and the player is asked to click on the new item(s) that were absent in the previous image. In Stampede, the player sees a special animal to be memorized. Then, a group of animals appear spinning on a computer screen, and the player has to click only on the animal that was originally presented. In the Whacky Animal training program, the player is shown animals that must be memorized; as the animals appear randomly on the screen, they must be clicked or touched before disappearing behind the screen. In Gone Fishing, the player has to fish by following the bobber and clicking on it.

In Duckneglect, the player is told to tap one or more objects belonging to a particular class (targets) that appear on the screen in a virtual scenario and to avoid distractions with the hand contralateral to the neglected space. The goal of this game is to guide players through visual search tasks.

MeMo is divided into two parts, and the first part involves memory. The second part involves flexibility and mental attention, which includes the following three activities: Arrows for processing speed, inhibitory control, and mental flexibility training; Tricky Cards for working memory training; and Jumping Squares for reaction anticipation and inhibitory control training.

BrainHQ includes 29 exercises that cover six areas of cognition, including memory, attention, speed, people skills, navigation, and intelligence.

Video game tasks involves two types of tasks to train attention: the flow task and the control task. These tasks have the same content, except that the flow task is designed by increasing the difficulty of the task according to the patients' skills and providing clear goals and quick feedback on the score. The task includes Square, Click Number, and Tower. In the Square task, patients must control a central blue square with the mouse and prevent red squares from entering the right, left, top, or bottom of the screen. If the squares coming towards the blue square are black, patients get points for hitting them. In the Click Number task, patients must click and delete disks in numerical order. In the Tower task, blocks of three colors are randomly stacked. Patients should click and delete the right, left, or center of the block based on its color as soon as possible. The removal time of all the blocks is calculated.

Caribbean Quest (CQ) is composed of five cognitive games: Scuba, Submarine, Wave, Pirate Delicatessen, and Squidditch. Scuba and Pirate Delicatessen are working memory games, Submarine and Wave are sustained attention games, and Squidditch is a selective attention game. Submarine and Wave are similar in cognitive tasks but different in terms of gameplay. Submarine is a static environment in which the player's only control is the selection of fish from the middle porthole, while the Wave allows the user to navigate an avatar in an ocean.

Brainastic includes 17 minigames, four of which are specific for attention. In Conveyor Belt, to learn versatility, you have to change the conveyor sorter and collect items with a specific color. In Spot the Difference, you need to find a unique insect to teach attention and the ability to filter information. In Film Collector, the film should be selected with a specific color or pattern to teach the immediate reaction to move objects. In Honey Haunters, to teach attention and the ability to filter information, you need to determine the correct number continuously over a short period.

RehAtt™ uses a 3D VR game environment to combine visual scanning training and multisensory stimulation. The hardware creates a virtual 3D world. The feeling of real touch and guidance is provided by a robotic pen and through vibrotactile feedback.

Video game therapy (Kinect Adventures and Kinect Sports) encompasses a wide range of physical activities. Kinect Adventures using full-body movement allows the player to participate in a variety of minigames. Kinect Sports is a collection of six sport simulations and eight small games designed to demonstrate the capabilities of Kinect motion. The six sports include bowling, boxing, athletics, table tennis, beach volleyball, and union football.

In Space Fortress, the player uses the joystick to steer a spaceship in an environment without friction and shoot the rocket into the space castle to destroy it; meanwhile, mines keep appearing on the screen.

VR-based games include the following: (a) Carnival Games: Monkey see Monkey (Wheel of Fortune, Strength Test, Court King, Granny Fling, Alley Ball, Ring Fling, Knockout Punch, Pig Race, Funnel Game, Crash Test Dummies, and Monkey) and (b) Kinect Adventures (Space Pop and River Rush).

### 3.5. Impact of Interventions on Attention Rehabilitation

In addition to attention measures, some studies also evaluated other cognitive domains such as executive functions, memory, and perception. Here, we only present the measures related to attention. Of 46 tools used in the studies, five used cross-modal oddball attention task [54, 56, 61, 73, 84], five game performance [42, 58, 66, 72, 80], three paced auditory serial additional test (PASAT) [53, 70, 77] and Symbol Digit Modalities Test (SDMT) [53, 70, 77], two Stroop test (ST) [53, 84], two the test of everyday attention (TEA) [62, 71], two the test of variables of attention (TOVA) [55, 64], and two the trail making test (TMT) [68, 70]. Other tools used once include EEG data analysis [52], ADHD-T questionnaire [52], CRSD-ant test questionnaire [52], CogState [55], Focused Spatial Attention Task [56], Distributed Spatial Attention Task [56, 57], Identification Test [57], digit cancellation task (D-CAT) [60], Digit Span Forward (DS-F) [60], Digit Span Backward (DS-B) [60], attentional blink [62], Toulouse-Pieron test (TPT) [63], attentional matrices [65], Vienna Test System (COG-S9) [66], negative priming task [84], Digit Symbol Substitution Test [68], Symbol Digit Modalities Test (SDMT) [70], continuous performance test X task (CPT-X) [70], Moss Attention Rating Scale (MARS) [70], neglect tests [75], Go/Nogo task [76], and Speed subtask [78]. These tools apply to different subdomains of attention.

Table 3 shows the evaluation results of each measure in each article. If no significant change is achieved in the measures, “No sig” is written in the result column. Also, in case of significant improvement, the word “Sig Improved” and for significant deterioration the word “Sig Deteriorated” are mentioned in the result column. In a total of 73 outcome measures related to attention, 42 measures did not have a significant result, 30 were significantly improved, one was significantly deteriorated, and four did not have any measures for attention evaluation. One article had conflicting results immediately after the intervention and on the three-month follow-up. Moreover, some articles did not evaluate specialized attention measures and, as such, are not listed in the table.

## 4. Discussion

This study identified and categorized serious computer games used for attention rehabilitation and assessed the effects of these games. Game categories were presented based on game genres and different platforms. To the best of our knowledge, this was the first review on this subject. Bogdanova et al. [5] conducted a systematic review somewhat closely related to this study, but they examined computerized rehabilitation programs, not computer games. On the other hand, they considered only attention deficits after acquired brain injury. Also, the study by Norman et al. was somewhat similar and examined the effectiveness of cognitive rehabilitation programs after acquired brain injury [5, 19]. Examples of the differences between serious games and traditional computer rehabilitation methods are the following: the ability to prevent boredom and monotony, increase motivation, and provide timely feedback and the ability to perform multitasking exercises, attractive visual interfaces, providing consistent content, and difficulty level according to the performance of players [85].

Upon examining 3937 articles, 30 articles and 22 unique games were included. Seven games were based on personal computer (PC) [42, 52, 55, 65, 70, 72, 77], five based on virtual and augmented reality [58, 59, 63, 75, 79], three based on web application [54, 61, 68, 69, 71, 73, 80, 84], two based on Kinect console [76], one based on Nintendo consoles [53, 60, 66], one based on mobile application [74], and three based on multiple platforms [56, 62, 64, 78].

Out of the 73 outcome measures, 30 showed significant improvement and only one showed significant deterioration [55]. Furthermore, 19 of the 26 studies that performed attention evaluation had at least one significant improvement in their measures [42, 52–54, 56, 58, 61–66, 72, 73, 75, 76, 78, 80, 84]. Therefore, most studies support the effects of serious game-based computer interventions for attention rehabilitation. Although these results are promising, several methodological issues need to be addressed in future studies to determine the effects of interventions more accurately. For example, most studies did not have an appropriate sample size to ensure the significance and validity of the results, so that 20 out of 30 studies had less than 20 participants in the intervention group [52–54, 57–63, 65, 66, 69, 71–73, 75–79]. Also, 5 studies were performed with 1 to 5 participants [58, 59, 65, 71, 79]. In addition, the type and severity of the disease causing the attention deficit may have affected the outcome. Participants' age can also affect the rehabilitation process. Moreover, the control groups are different across studies; 11 studies provided routine care to the control group [53–55, 61–63, 66, 68, 70, 72, 77], whereas other studies included video, cartoon, and other active control groups. One-third of the studies did not have a control group at all, and out of 12 RCT studies, six studies include an active control group for comparison [57, 60, 73, 74, 78, 84]. Evidently, considering routine care as a control group will increase the risk of bias; that is, the positive effect of the intervention may not be due to increased physical, group, and social activity [86]. Therefore, it is recommended that future studies include active control groups.

The lack of long-term follow-up was another problem. As observed in the study by Ballesteros et al. [54], the results demonstrated a significant improvement immediately after the intervention but a nonsignificant result on the three-month follow-up. This could also be true in the case of other studies; therefore, follow-up should be performed at longer intervals to evaluate the validity and persistence of the result.

The reviewed studies used different tools to measure attention and its subdomains. Some tools can be applied to all cognitive domains, while some are specific to the domain of attention or its subdomains. There is no standard for measuring attention, and this necessitates a comprehensive and appropriate standard for this purpose. Another important point in game design is the role of scientific teams in game design. Other studies emphasize the participation of therapists and scientific teams in the design process [5, 36]. The benefits of being a therapist along with the use of these tools include higher patient motivation, better feedback for the physician, and better management and control of the device according to the patient's condition [31]. Most of the reviewed studies did not mention the exact process or the team involved in game development. The development of standard games with the participation of various scientific groups and stakeholders seems to be necessary for attention and its subcategories, and standard methods must be developed to use and evaluate the attention rehabilitation process. Unfortunately, the increasing growth of programmers and commercial companies producing games and medical equipment, without the use of specialized scientific groups, poses many dangers and harms to the health of people in the community. It can be seen that there are many commercial tools that are unjustifiably advertised in the market to improve various mental skills. There is a need to take action to solve this problem [31]. For example, in all tools or games in the field of public health, basic criteria can be defined, both based on the interface and usability and based on their content. Extensive clinical evaluation may be mandatory for all instruments to obtain a license to use. Also, systematic review studies can be helpful for this purpose.

Due to the insufficient RCT studies and the existence of various measures, it was not possible to perform a meta-analysis on the data. Moreover, the control groups greatly differed. For many games, no clinical trial was performed. There were also many articles on general cognitive rehabilitation in which attention rehabilitation was a subscale. Thus, the search was very comprehensive and we tried to overcome this limitation by conducting a more comprehensive search and excluding irrelevant articles.

As mentioned in the introduction, attention deficit disorder is usually associated with other cognitive impairments, and each of these deficits may have a positive or negative effect on the other. Perhaps it would have been better if this study had evaluated all these cognitive deficits together, but due to their great complexity and diversity, this was not possible in the study. Therefore, because attention deficit is fundamental and rehabilitation and improvement can help promote other cognitive deficits and reduce disability, we focused only on the area of attention deficit [5]. The fundamentality of attention skills means that, for example, attention skills support patients' visual and spatial perceptual ability to help predict next action. The ability of working memory is also required to temporarily store information to perform actions. On the other hand, maintaining patients' attention is a prerequisite for using their working memory ability [87].

## 5. Conclusions

In this systematic review, we reported game-based computer technologies for attention rehabilitation and assessed the effects of these games. In 19 out of 26 articles measuring the criterion of attention, at least one significant improvement was reported; therefore, it can be concluded that games created for attention rehabilitation are effective and patients and therapists can use the significant benefits of these games to rehabilitate attention deficits. However, to prevent bias, factors such as the variety of criteria and differences in their measurement, small sample size, lack of accurate evaluations of clinical trials on games, absence of scientific teams, lack of long-term follow-up, the existence of different control groups, and not including active groups for comparison should be considered when designing, developing, and using game-based systems. On the other, most of the games found in our study are presented on PC and virtual reality platforms, and for the benefits of portability and continuity of use, we suggest that more games be made for portable platforms such as mobile and tablet.

## Figures and Tables

**Table 1:** Summary of the included studies.

Num	Author. (year)	Journal	Country	Study design	Intervention (game)	Control group	Target group	Participants	Mean age	Session number, frequency, duration	Result
1	Mercado et al. [52] (2020)	Journal on Multimodal User Interfaces	Mexico	Non-RCT (quasi-experimental)	A BCI video game (FarmerKeeper)	Cartoons	Children with autism	26 (IG = 13; CG = 13)	8.0 ± 3.07	13 sessions, 15 min, 3 blocks around 4 min each	All measures of attention, sustained attention, and attentional control in all children show improvement
2	De Giglio et al. [53] (2015)	Neurorehabilitation and Neural Repair	Italy	Pilot RCT	A home-based cognitive rehabilitation program (Dr. Kawashima's Brain Training)	Waitlist (usual care)	Multiple sclerosis	35 (IG = 18; CG = 17)	43.9 ± 8.4	40 sessions, 8 consecutive weeks, 30 min/d, 5 d/wk	Significant improvement in the effect of DKBT on ST, SDMT, and some MSQoL subscales was observed. Improvements were also observed in the cognitive subscales MFIS and PASAT, but this improvement was not significant
3	Montani et al. [42] (2014)	Frontiers in Psychology	Italy	Uncontrolled before and after clinical trial	A new adaptive video game Labyrinth (“diamond task” and “snake task”)	—	Traumatic brain injury (TBI)	20	20.8 ± 1.5	14 sessions, 40 min, 2 weeks	The results confirmed the strengthening of cognitive abilities by the game, especially the improvement of attentional control during the game
4	Ballesteros et al. [54] (2015)	Frontiers in Aging Neuroscience	Spain	RCT	Nonaction video game training (Lumosity)	Usual care	Elderly	28 (IG = 17; CG = 11)	69.0 ± 5.53	20 sessions, 1 hour, 10–12 weeks	Processing speed, attention, and visual recognition memory as well as two dimensions of subjective well-being showed significant improvement
5	Boivin et al. [55] (2016)	AIDS Research and Human Retroviruses	Ugandan+USA	RCT	Computerized cognitive rehabilitation training (Captain's Log)	Usual care	Children with HIV	157 (CCRT = 51; limited CCRT = 52; CG = 54)	8.9 ± 1.86	24 sessions, 1 hour, 3 days per week	The overall KABC-II mental processing index, knowledge, and planning improved significantly compared to passive controls. Significant improvements were seen in CogState Groton maze chasing, card detection, and learning in both CCRT arms. However, in other CogState memory or attention measures, TOVA, BRIEF, and CBCL were there any different in the arms
6	Franceschini et al. [56] (2013)	Current Biology	Italy	Uncontrolled before and after clinical trial	Video games	—	Dyslexia	20	9.83 ± 17.25	9 sessions, 80 min per day	The results showed that only action video games helped to increase children's reading speed, attention abilities, and skills
7	Guimaraes et al. [57] (2018)	Journal of Physical Education and Sport	Brazil	RCT	Active video game-based (AVG) physical activity program	Aerobic exercise program	Elderly	27 (IG = 13; CG = 14)	60.4 ± 3.8	36 sessions, 3 times a week, 12 weeks	In the AVG group, only executive function and delayed memory improved, and in the aerobic group, visual attention, executive function, delayed memory, short-term memory, and overall cognition improved. However, we did not find significant differences between groups in the performance of cognitive tests. This suggests that the benefits of AVG exercise may be similar to those of regular aerobic exercise
8	Alqithami et al. [58] (2019)	Healthcare	Saudi Arabia	Single-subject design (non RCT)	Augmented reality game (AR-Therapist)	—	ADHD	1	—	Depend on child performance and his engagement level, 10 trials of 1 minute each	The patient performs better in selecting a predetermined object, which indicates a positive performance index
9	Boletsis et al. [59] (2016)	International Journal of Serious Games	Norway	Uncontrolled clinical trial	Augmented reality cube game (CogARC system)	—	Dementia	5	67.6 ± 5.77	To complete two levels of each minigame approximately 25–30 minutes in total	The iGEQ test showed improvement in positive effect, immersion, and challenge. However, some values indicate specific problems in several small games. Also, the usability score by the SUS test in CogARC was higher
10	Nouchi et al. [60] (2012)	PloS One	Japan	RCT	A popular brain training game (Brain Age)	A popular puzzle game (Tetris)	Elderly	32 (IG = 16; CG = 16)	69.08 ± 2.44	20 sessions, 15 minutes per day, 5 days per week, 4 weeks	The results showed that in all measures of executive function, TMT-B, and two measures of processing speed, the intervention game had a better result than the Tetris game as control. However, there is no significant difference between the effect of Brain Age and Tetris in measuring global cognitive status and all attention measures
11	Ballesteros et al. [61] (2014)	Frontiers in Aging Neuroscience	Spain	RCT	20 nonaction video games (Lumosity)	Usual care	Elderly	40 (IG = 20; CG = 20)	69.0 ± 5.53	20 sessions, 1 hour, 10–12 weeks	Attention, processing speed, immediate, delayed visual recognition memory, and the two dimensions of the well-being scale (affection and assertiveness) showed significant improvement
12	Vakili et al. [62] (2016)	Cogent Psychology	Australia	RCT	Action video game (Medal of Honor: Rising Sun (MoHRS))	Waitlist (usual care)	Traumatic brain injury (TBI)	26 (IG = 15; CG = 11)	28.57 ± 8.10	8 sessions, 2 hours, 8 weeks	The intervention resulted in a significant improvement in game performance and an effect of lag in both groups showed by the attentional blink task. The detection of the second target at all-time lags showed great progress for the intervention group. Also, the attention training group showed a significant improvement in map search (2 min), but this improvement was not significant in the other two TEA methods. In contrast, a significant decrease was observed in the TAU group. No improvement was observed in the BRIEF-A (executive performance) or GSES (self-efficacy) scales
13	Gamito et al. [63] (2017)	Disability and Rehabilitation	Portugal	RCT	A virtual reality-based serious game application	Waitlist (usual care)	Stroke	20 (IG = 10; CG = 10)	55.0 ± 13.5	8-18 sessions, 2-3 sessions per week, 1 hour, 4–6 weeks	Unlike the control group, in the intervention group, a significant improvement in patients' WMS scores and efficiency was observed. Significant interaction was also seen on work efficiency of sustained attention
14	Giordani et al. [64] (2015)	Global Mental Health	Uganda	Uncontrolled before and after clinical trial	A computer-based training platform (Brain Powered Games (BPG) package)	—	At-risk African children	33	8.55 ± 2.29	24 sessions, 45 min, 3 days per week, 2 months	Attention measurements (TOVA omissions), processing speed (TOVA response time), basic visuomotor tracking speed (GMLT chase test), and problem solving (GMLT learning test) were significantly improved. In contrast, TOVA percent commission errors and KABC-II nonverbal index composite score did not significantly improve as a result of BPG training
15	Mainetti et al. [65] (2013)	Technology and Health Care	Italy	A case series (non-RCT)	A set of designed games (Duckneglect)	—	Neglect disorder	1	—	20 sessions, 30 minutes, 5 days a week, 1 month	Peripersonal neglect on the line bisection task, MMSE, and the attentional matrices showed a significant improvement. Despite the improvement in postsession test performance, this improvement was not stable until the end of the rehabilitation period and a five-month follow-up showed that the patient remained stable
16	Chen et al. [66] (2012)	Turkish Online Journal of Educational Technology (TOJET)	Taiwan+Canada	Non-RCT	Somatosensory video game trainings (three games)	Usual care	Elderly	35 (IG: 4 weeks = 8; IG: 8 weeks = 10; CG: 4 weeks = 8; CG: 4 weeks = 9)	79.09 ± 6.61	12-24 sessions, 30 min, 3 times per week, 4 and 8 weeks	In most participants, after 8 weeks of follow-up, selective attention in immediate effect, carry-forward effects, and overall effect improved significantly and did not show rapid improvement overall. In the end, they concluded that the use of somatosensory video games to promote the selective attention of the elderly with disabilities is a good approach
17	Ballesteros et al. [67] (2017)	Frontiers in Aging Neuroscience	Spain	RCT	Nonaction video games from Lumosity	An active control group with simulation strategy games	Elderly	55 (IG = 30; CG = 25)	65.46 ± 5.07	16 session, 40–50 min, 10–12 weeks	Contrary to the measurement of selective attention and working memory, a significant improvement was seen in the performance of participants in the training sessions While both groups progressed similarly in the Corsi block task, a marginal training effect was observed for the N-back task, and no progress was observed for the Stroop task in the experimental group
18	Robert et al. [68] (2020)	Journal of Medical Internet Research	France	RCT	MeMo (Memory Motivation) web application	Usual care	Neurocognitive disorders	46 (IG = 25; CG = 21)	79.4 ± 6.8	48 session, 30 min, 4 per week, 12 and 24 weeks	Attention tests (trial making test A and correct digit symbol substitution test items), and the apathy inventory (AI) showed significant differences between MeMo and nonactive MeMo groups
19	Sharma et al. [69] (2017)	Disability and rehabilitation	Canada	A case series (non-RCT)	The cognitive training program (brain HQTM)	—	Moderate-severe brain injury	10	43.7 ± 16.14	60 sessions, 60 min, 5 days per week, 12 weeks	Patients' adherence to the intervention was moderate, and there was 70% patient retention
20	Yoshida et al. [70] (2018)	Neurorehabilitation	Japan	Pilot RCT	Two types of video game tasks: a flow task and a control task	Usual care	Traumatic brain injury (TBI)	20	41.7 ± 9.37	40 sessions, 20 min, 2 in a day, 4 weeks	—
21	Zickefoose et al. [71] (2013)	Brain injury	USA	Single-subject design (non-RCT)	Attention process Training-3 (APT-3) and Lumosity brain games (Birdwatching, Monster Garden, Playing Koi, Rotation Matrix, and Top Chimp)	—	Traumatic brain injury (TBI)	4	42.75 ± 7.80	20 sessions, 30 min, 1 month	Although participants made significant progress in both interventions, there was a limited generalization
22	Macoun et al. [72] (2020)	Journal of Autism and Developmental Disorders	Canada	Non-RCT	A game-based cognitive training program (Caribbean Quest)	Waitlist (usual care)	Children with autism	20 (IG = 11; CG = 9)	8.64 ± 1.74	24 sessions, 30 min, approximately 3 times per week, 8–10 weeks	Executive function or attention performance measures: the error rate in the intervention group was significantly lower compared to the control group. For KiTAP “owls” (divided attention) or “ghost ball” (sustained attention) tasks and WISC-IV spatial span or digit span tasks, no differences were observed compared to before the intervention. There was a significant difference in errors between the intervention and control groups in the visual-spatial WM task of “colored boxes.” Academic fluency: fewer errors in the intervention group than the control group were significant. There was no difference in oral reading fluency in the intervention group
23	Mayas et al. [73] (2014)	PLOS One	Spain+Australia	RCT	10 video games selected from Lumosity	Discussion meetings about general topics related to aging	Elderly	27 (IG = 15; CG = 12)	68.6 ± 5.45	20 sessions, 1 hour, 10–12 weeks	Significant increase in alertness and decrease in distraction were observed in the experimental group, but no change was observed in the control group
24	Yu et al. [74] (2021)	International Journal of Environmental Research and Public Health	China	RCT	Brainastic computerized cognitive training (CCT)	Video watching on history, art, literature, and science+physical exercise	Elderly	232 (IG1: multidomain CCT + PE = 117; IG2: two-domain CCT + PE = 116; video watching+PE = 114)	64.2 ± 6.4	IG1, IG2: 24 sessions, 1 hour PE + 30 min Brainastic CCT session	The improvement in frailty status, learning ability, and verbal memory ability was quite visible in the participants in the intervention groups (multi-/two-domain CCT+PE) compared to the control participants. Multidomain CCT did not perform better in improving frailty status or cognitive function than two-domain CCT
25	Fordell et al. [75] (2016)	Topics in stroke rehabilitation	Sweden	Uncontrolled before and after clinical trial	Multisensory stimulation in virtual reality (RehAtt)	—	Chronic neglect after stroke	15	72.8 ± 5.7	15 sessions, 1 hour, 3 times per week, 5 weeks	Improvement due to training was seen in the baking tray task, star cancellation test, and extinction test. Fewer missed goals in Posner's task were improved. CBS continued to show improvements in daily activities, both immediately after training and after 6 months of follow-up
26	Straudi et al. [76] (2017)	BMC neurology	Italy	Exploratory, pilot RCT	Preselected games (Kinect Adventures and Kinect Sports)	A balance platform therapy (BPT) by Biodex Medical Systems	Traumatic brain injury (TBI)	21 (IG:VGT = 11; CG:BPT = 8)	36.0 ± 12.0	18 sessions, 1 hour, 3 per week, 6 weeks	CB&M scores improved in both groups, but only UBS and TUG increased in the VGT group. Also, in the VGT group, selective attention was significantly improved
27	Janssen et al. [77] (2014)	Journal of Clinical and Experimental Neuropsychology	USA	Pilot RCT	Hybrid-variable priority training (HVT) program (Space Fortress game)	Waitlist	Multiple sclerosis	28 (IG = 14; CG = 14)	47.18 ± 7.6	20 sessions, 1 hour (part task training: 10 sessions, variable priority training: 10 sessions)	Except for the WTAR test, other tests (BDI-II, PASAT, SDMT, SRT, LTS, CLTR, and WLG) did not show significant improvement in the intervention group. There was evidence in improving skill acquisition and feasibility of the intervention, but there was no evidence of widespread transfer to cognitive function tasks. However, an improvement in spatial short-term memory was seen in participants. Also, attention and executive function did not show significant improvement, and verbal memory showed a higher rate in the control group. The visual memory in the intervention group showed a significant improvement. No significant change was seen in the transition to long-term spatial memory measurements by the 10/36 spatial recall delay version. Also, no significant results were observed for higher-order functions, which are measured by the demand for verbal fluency of the word list generation task
28	Belchior et al. [78] (2013)	Computers in Human Behaviour	Canada+USA	RCT	Medal of Honor: Rising Sun (MoHRS)	A placebo control arcade game (Tetris), useful field of view (UFOV) training program, a usual care control group	Elderly	58 (IG: MOH = 14; CG:Tetris = 16; CG:UFOV = 15; CG:no contact = 13)	74.7 ± 6.4	6 sessions, 90 min, 2–3 weeks	Significant improvement was seen in UFOV compared to game groups. On the other hand, a significant improvement was observed in all three intervention groups compared to the noncontact control group. Also, contrary to the findings observed in the younger adults, there was no difference between the two game states
29	Muneer et al. [79] (2015)	Disability, CBR, and inclusive development	India	Uncontrolled before and after clinical trial pilot	Virtual reality-based games: Carnival games, Kinect Adventures	—	Children with developmental disabilities	5	5.2 ± 1.09	4-6 sessions, 20-30 min, 1 month	Significant improvements in specific motor skills and cognitive, social, and emotional skills were seen in children. No withdrawal of children was performed in any of the skills from different areas
30	Castro-Rojas [80] (2018)	Gerontechnology	Denmark	Uncontrolled before and after clinical trial	A web-based game application (Lumosity)	—	Elderly	51	67.10 ± 5.40	3 sessions, 2.5 hours, 6 weeks	The performance of online cognitive games in participants was improved by participants with repetitive practices. Unlike factors such as age, education, and a positive attitude towards technology, the significant effect of the number of game times on performance improvement was statistically quite clear

ADHD: attention-deficit hyperactivity disorder; HIV: human immunodeficiency virus; RCT: randomized controlled trial; BCI: brain-computer interfaces.

**Table 2:** Characteristics of the games used in the included articles.

Game	Genre	Developer	Released date	Platform	Language	Description	Paper number
FarmerKeeper	Action (platformers)	Mercado et al.	2018	PC based	English	The game story unfolds on a farm. The goal of the game is to maintain children's attention above a threshold to control a runner who is seeking for lost farm animals to take them back to their pens	1
Brain Age (Dr. Kawashima's Brain Training)	Casual (puzzle)	Commercial (Nintendo)	2020	Wii U, Nintendo DS	Italian	Puzzles and minigames to strengthen the player's memory and concentration skills	2, 10, 16
Labyrinth	Casual (board game)	Montani et al.	2014	PC based	English	A little man moves along a maze to reach a goal. The game character is controlled by the gamer through a joystick	3
Lumosity	Casual (puzzle)	Commercial (Lomus Lab)	2021	Web based	English, Spanish, German, French	These video games include 50 games, 10 of which are specifically related to attention (Assist Ants, Feel the Beat, Skyrise, Eagle Eye, Playing Koi, Trouble Brewing, Train of Thought, Lost in Migration, Star Search)	4, 11, 17, 21, 23, 30
Captain's Log	Action (platformers)	Sandford et al.	1988	PC based	English	BrainTrain's software products are designed for decision support, education, research, and maintaining a healthy lifestyle purpose	5
Rayman Raving Rabbids	Action role-playing game (RPG)	Commercial (Ubisoft)	2006	Wii, PlayStation 2, Microsoft Windows, Xbox 360	English	The game features two different modes of play—“story mode” and “score mode.” In the story mode, the game follows fifteen days of Rayman's imprisonment by the Rabbids. Each day, Rayman must complete at least three trials, followed by one special “boss trial,” such as a first-person rail shooter using plungers or a racing game in which the player controls a warthog and uses a flyswatter as a riding crop. Minigames fall into one of four categories: Bunny Hunt, Sports, Challenges, and “Shake your Booty!” dancing	6
Kinect Sports Ultimate Collection: athletics, bowling, boxing, skiing, soccer, tennis, and table tennis and minigames	Simulation (sport)	Commercial (Rare)	2010	Xbox 360 Kinect	English	Includes 13 sports: basketball, soccer, American football, bowling, beach volleyball, table tennis, boxing, golf, tennis, skiing, darts, and baseball	7
AR-Therapist	Simulation (sport)	Alqithami et al.	2019	Microsoft HoloLens emulator	English	A simulated augmented reality environment using a simple game	8
CogARC system	Casual (puzzle, word game)	Boletsis et al.	2016	AR on a tablet PC	English	CogARC is a serious game for cognitive training and screening, utilizing an interaction technique based on augmented reality (AR) and the manipulation of tangible, physical objects (cubes). The game is a collection of cognitive minigames of preventative nature	9
Medal of Honor: Rising Sun (MoHRS)	Action (first-person shooters)	Commercial (EA Games)	2003	GameCube, PlayStation 2, Xbox	English	Rising Sun is set in World War II during the Pacific War	12, 28
A virtual reality-based serious game application	Simulation (process)	Gamito et al.	2015	VR on PC	English	Comprised several daily life activities that were devised to train cognitive functions such as buying several items, finding the way to the minimarket, finding a virtual character dressed in yellow, and recognition of outdoor advertisements)	13
Brain Powered Games (BPG) package	Casual (puzzle, word game)	Giordani et al.	2015	PC and smartphone with multiple platforms	English	A computer-based training platform	14
Duckneglect	Casual (puzzle, word game)	Mainetti et al.	2013	PC based	English	A set of specifically designed games that is based on three key elements: games to guide rehabilitation, hands-free motion tracking, and the display of mirror images	15
MeMo (Memory Motivation) web app	Action (platform)	Scientific group (CoBTeK research team in the Nice Memory Center at the Institute Claude Pompidou)	2015	Web based, mobile application	French, English, Italian	MeMo is divided into two parts. The first part involves memory, which includes the following three activities: “recognition” for visual memory training, “MeMo quiz” for working memory training, and “faces” for associative memory training. The second part involves mental flexibility/attention, which includes the following three activities: “Arrows” for processing speed, inhibitory control, and mental flexibility training; “Tricky Cards” for working memory training; and “Jumping Squares” for reaction anticipation and inhibitory control training	18
BrainHQ	Casual (puzzle, word game)	Scientific group & commercial (Posit Science)	2017	Web based, mobile application	Multiple languages: English, German, Japanese, etc.	BrainHQ is comprised of 29 exercises, which target six areas of cognition: memory, attention, speed, people skills, navigation, and intelligence	19
A video game task	Casual (puzzle, word game)	Yoshida et al.	2014	PC based	English	Two types of video game tasks for attentional training; one is a flow task and the other a control task. The task includes Square, Click Number, and Tower	20
Caribbean Quest (CQ)	Multiple genre: Scuba: platformers, Pirate Deli: simulation (cooking), Submarine: casual (puzzle), Wave: action (platformers), Squidditch: casual (puzzle))	Scientific group (University of Victoria, funded as a special project by Kids Brain Health Network (NCE)	2012	PC based	English	The CQ consists of five hierarchically structured, self-adjusting minigames that train WM, inhibitory control, selective attention, and sustained attention	22
Brainastic	Casual (puzzle)	Commercial (Mindvivid Limited)	2016	Mobile phones	Chinese	Brainastic is an online application for cognitive training through video games and is performed on a tablet with each game targeting one of the five domains including 17 minigames (Forest of Memory, Catch the Star, Colored Light Bulbs, Master of Oriental Stitch, Conveyor Belt, Spot the Difference, Film Collector, Honey Haunters, Conquer the Ice, From Small to Big, Switch and Match, Piet Mondrian Mansion, Color or Shape, Save the Daruma, Pairing Detective, Fixing Pixels, Dance in the Rain	24
RehAtt™	Casual (puzzle)	Fordell et al.	2016	VR on PC based	English	The hardware creates a virtual 3D world. The robotic pen gives a guiding force feedback and a realistic touch sensation through vibrotactile feedback. The subject can see the robotic pen as a stick coming out of the screen. 3D objects can be moved, rotated, and manipulated, giving a sense of depth	25
Video game therapy: “Kinect Adventures” and “Kinect Sports”	Multiple: simulation (sport, adventure, action, Exergaming)	Commercial (Microsoft Game Studios+Rare)	2010	Xbox 360 Kinect	English	Preselected games were chosen from “Kinect Adventures” and “Kinect Sports” that encompassed a wide range of motor activities in a standing position	26
Space Fortress game	Action (shooter)	Emanuel Donchin (Daniel Gopher's laboratory)	1984	BBC Micro (PC)	English	The player, using a joystick, navigates their spaceship in a frictionless environment, shooting missiles at the Space Fortress to destroy it, while simultaneously monitoring and collecting bonus points that appear at the bottom of the screen and constantly dealing with diamond-shaped foe or friend mines that appear on the screen	27
Virtual reality-based games: (a) Carnival Games: Monkey see Monkey do and (b) Kinect Adventures	(a) Multiple: action, role-playing game (RPG), and party (b) Multiple: adventure and simulation (sport)	Commercial ((a) Cat Daddy Games and (b) Microsoft Game Studios)	(a) 2011 (b) 2010	VR on Xbox 360 Kinect	English	(a) Carnival Games: Wheel of Fortune, Strength Test, Court King, Granny Fling, Alley Ball, Ring Fling, Knockout Punch, Pig Race, Funnel Game, Crash Test Dummies, and Monkey see Monkey do (b) Kinect Adventures: Space Pop and River Rush	29

**Table 3:** Attention measures and results.

Num	Assessment tools	Attention subdomain	Index	Result		Ref
1	EEG data	General	Level of attention (% of time with an attention)	Sig improved		[52]
	EEG data		Number of changes between threshold (^∗^a lower number of changes is better)	Sig improved		
	EEG data		Length of attention block (seconds)	No sig		
	ADHD-T questionnaire	General	Distracted frequency(^∗^a lower number is better)	Sig improved		
	CRSD-ant test questionnaire	Sustained attention	Average score of sustained attention	Sig improved		
2	Stroop test (ST)	Sustained attention	Mean (SD) score	Sig improved		[53]
	Paced auditory serial additional test (PASAT)	Sustained attention	Mean (SD) score	No sig		
	Symbol Digit Modalities Test (SDMT)	Sustained attention	Mean (SD) score	Sig improved		
3	Game performance	General	Task difficulty level	Sig improved		[42]
	Game performance	General	Time limit	Sig improved		
	Game dual task performance	Divided attention	Diamond time (DT)	Sig improved		
	Game task switching performance	Alternating attention	Diamond time (DT)	Sig improved		
4	Cross-modal oddball attention task	Visual, spatial, focused, divided, selective, and transient attention	Distraction score	Post	Sig improved	[54]
				3-month follow-up	No sig	
			Alertness score	Post	Sig improved	
				3-month follow-up	No sig	
5	The test of variables of attention (TOVA)	Sustained attention	Score	No sig		[55]
	CogState	General	Simple reaction time: playing card turning	No sig		
	CogState	General	Choice reaction time: red playing card turning log msec	No sig		
	CogState	General	Maze chase correct moves per second	Sig deteriorated		
6	Focused spatial attention task	Focused attention	Mean accuracy (SD)	Sig improved		[56]
	Distributed spatial attention task	Divided attention	Mean accuracy (SD)	Sig improved		
	Cross-modal oddball attention task	Visual, spatial, focused, divided, selective, and transient attention	First cue-target interval RT (ms)	No sig		
			Second cue-target interval RT (ms)	No sig		
			First cue-target interval accuracy	No sig		
			Second cue-target interval accuracy	No sig		
7	Identification test	Visual attention	Accuracy	No sig		[57]
			Speed perf. (ms)	No sig		
8	Game performance	General	Correct tries, number of omission errors, number of commission errors, number of uncompleted tries, correct response times, try time, engagement factor, inattention factor, impulsivity factor, error factor, and correct response factor	Sig improved		[58]
9	—	—	—	—		[59]
10	Digit cancellation task (D-CAT)	General	Mean (SD) score	No sig		[60]
	Digit Span Forward (DS-F)	General	Mean (SD) score	No sig		
	Digit Span Backward (DS-B)	General	Mean (SD) score	No sig		
11	Cross-modal oddball attention task	Auditory, visual, focused, selective, divided, and transient attention	Distraction (mean (SD) score)	Sig improved		[61]
			Alertness (mean (SD) score)	Sig improved		
12	The test of everyday attention (TEA)	Visual, selective attention	Map search	Sig improved		[62]
		Visual, selective attention	Telephone search	No sig		
		Alternating attention	Visual elevator (number correct)	Sig improved		
		Sustained attention	Elevator counting	No sig		
		Sustained attention	Telephone search (dual task decrement)	No sig		
		Auditory, focused attention	Elevator counting with distraction	Sig improved		
		Auditory, alternating attention	Elevator counting with reversal	No sig		
	Attentional blink	Visual, selective, focused, alternating, transient attention	Score	No sig		
13	Toulouse-Pieron test (TPT)	Sustained attention	Score	Sig improved		[63]
14	The test of variables of attention (TOVA)	Sustained attention	Percent omission errors, percent commission errors, response time (ms), response time variability (ms)	Sig improved		[64]
15	Attentional matrices	Visual, auditory, focused attention	Score	Sig improved		[65]
16	Vienna Test System (COG-S9)	Selective attention	Score	—		[66]
	Game performance	General	Sum reactions, percentage incorrect reaction, sum correct reaction, sum incorrect reaction, mean time correct reactions, mean time incorrect reactions, sum hits, sum correct rejections	Sig improved		
17	Cross-modal oddball attention task	Selective attention (distraction & alertness)	Mean differences in reaction time	No sig		[84]
	Stroop test (ST)	Effortful inhibitory control	Mean reaction time	No sig		
	Negative priming task	Automatic passive inhibition	Mean reaction time	No sig		
18	Trail making test (TMT)	General	Mean (SD) score	No sig		[68]
	Digit symbol substitution test	General	Mean (SD) score	No sig		
19	—	—	—	—		[69]
20	Symbol Digit Modalities Test (SDMT)	General	Mean (SD) score	No sig		[70]
	Trail making test (TMT)	General	Mean (SD) score	No sig		
	Paced auditory serial additional test (PASAT)	General	Mean (SD) score	No sig		
	Continuous Performance Test X task (CPT-X)	General	Mean (SD) score	No sig		
	Moss Attention Rating Scale (MARS)	General	Mean (SD) score	No sig		
21	Test of everyday attention (TEA)	Visual, selective attention	Map search	No sig		[71]
		Visual, selective attention	Telephone search	No sig		
		Alternating attention	Visual elevator (number correct)	No sig		
		Sustained attention	Elevator counting	No sig		
		Sustained attention	Telephone search (dual task decrement)	No sig		
		Auditory, focused attention	Elevator counting with distraction	No sig		
		Auditory, alternating attention	Elevator counting with reversal	No sig		
22	Test of attentional performance Children's version (KiTAP): game performance	Ghost's ball: sustained attention	Mean difference in errors	No sig		[72]
		Sad/happy ghost: selective attention	Mean difference in errors	Sig improved		
		Owls: divided attention	Mean difference in errors	No sig		
23	Cross-modal oddball attention task	Auditory, visual, focused, selective, divided, and transient attention	Mean differences in reaction time	Sig improved		[73]
24	—	—	—	—		[74]
25	Neglect tests (VR-test battery)	Visual spatial attention	Mean score	Sig improved		[75]
26	Go/Nogo task	Selective visual attention	Mean (SD) score	Sig improved		[76]
27	Paced auditory serial additional test (PASAT)	General	Mean (SD) score	No sig		[77]
	Symbol Digit Modalities Test (SDMT)	General	Mean (SD) score	No sig		
28	Speed subtask	Visual divided, selective attention	Mean (SD) score	Sig improved		[78]
29	—	—	—	—		[79]
30	Game performance (Lumosity Performance Index (LPI))	General	Mean (SD) score	Sig improved		[80]

EEG: electroencephalogram; Sig improved: significantly improved; No sig: no significant change; Sig deteriorated: significantly deteriorated.

## Data Availability

No data were used to support this study.

## Abbreviations

ADHD: Attention-deficit hyperactivity disorder
HIV: Human immunodeficiency virus
RCT: Randomized controlled trial
BCI: Brain-computer interfaces
EEG: Electroencephalogram.

## Supplementary Materials

Supplementary Materials Search strategy and process of screening of studies.
