# Supplementary material for: A Systematic Review on Serious Games in Attention Rehabilitation and Their Effects
Source: Behav Neurol. 2022 Feb 26;2022:2017975. doi: 10.1155/2022/2017975 (PMC8898139; doi:10.1155/2022/2017975)
Supplement: Supplementary Materials — Search strategy and process of screening of studies. [file 2017975.f1.docx]

Table S1- Concepts and keywords identified for search

|  | **Concept1** | | **Concept2** | | **Composition of subcategories of concept2** | | | |
| --- | --- | --- | --- | --- | --- | --- | --- | --- |
|  | Serious game | | Cognitive rehabilitation | | Cognitive dysfunction | | rehabilitation | |
| **MeSH** | Video games | | Cognitive Remediation, Cognitive Behavioral Therapy | | Cognitive Dysfunction | | Rehabilitation  Therapeutics | |
| **EmTree** | Video game | | Cognitive Remediation Therapy, Cognitive Rehabilitation, Cognitive Therapy, NeuroRehabilitation | | Cognitive defect | | Rehabilitation  Therapy | |
| **Keywords** | ***Main terms*** | ***Search*** | ***Main terms*** | ***Search*** | ***Main terms*** | ***Search*** | ***Main terms*** | ***Search*** |
|  | computer games  video games  videogame  online game*  on-line game*  applied games  serious games  gamification  simulation games  Virtual reality games  Augmented reality games  Mobile game*  television game*  TV game* | computer game*  video game*  videogame*  online game*  on-line game*  applied game*  serious game*  gamification  simulation game*  Virtual game*  Virtual reality game*  VR game*  Augmented reality game*  AR game*  Mobile game*  Television game*  TV game* | Cognitive training  Cognitive rehabilitation  Cognitive-behavioral therapy  Cognitive therapy  Brain training  Cognitive enhancement therapy  Cognitive remediation  Memory training  Attention training  Reasoning training  Information processing speed rehabilitation  Executive function  cognitive functioning  Problem solving  Perceptual intervention  Spatial intervention  Visuoperceptual intervention  Neurorehabilitation  Visual processing rehabilitation  Information processing rehabilitation | Cognitive train*  Cognitive rehabilitation  Cognitive-behavioral therap*  Cognitive therap*  Cognitive enhancement therap*  Cognitive remediat*  Memory train*  Memory rehabilitation  Memory therap*  Memory remediation  Attention train*  Attention rehabilitation  Attention therap*  Attention remediation  Reasoning train*  Reasoning rehabilitation  Reasoning therap*  Reasoning remediation  Executive function* train*  Executive function* rehabilitation  Executive function* therap*  Executive function* remediation  Process* speed train*  Process* speed rehabilitation  Process* speed therap*  Process* speed remediation  Problem solv* train*  Problem solv* rehabilitation  Problem solv* therap*  Problem solv* remediation  Percept* train*  Percept* rehabilitation  Percept* therap*  Percept* remediation  Spatial train*  Spatial rehabilitation  Spatial therap*  Spatial remediation  Brain train*  Brain rehabilitation  Brain therap*  Brain remediation  Neurorehabilitation  Neurotraining | Cognit*  Neurocognit*  Executive function  Reasoning  Orientation  Perception  Thinking  Attitudes  Attention  Reasoning  Problem solving  Thinking  Visual-spatial | Cognit*  Executive function*  Reasoning  Orientation  Perception*  Thinking  attitude*  Problem solv*  Process* speed*  Attention  Spatial  Visual-spatial  attention | Remediation  Reconstruction  Rehabilitation  Training  Therapy  Therapeutic  Treatment  Readaptation | Remediat*  Reconstruction*  Rehabilitation*  Train*  Therap*  Treatment*  Readaptation* |

Table S2- Search strategy in scientific databases

| 1. **PubMed search strategy:** |
| --- |
| (Video games[MeSH Terms] OR computer game*[Title/Abstract] OR video game*[Title/Abstract] OR videogame*[Title/Abstract] OR online game*[Title/Abstract] OR applied game*[Title/Abstract] OR serious game*[Title/Abstract] OR gamification[Title/Abstract] OR simulation game*[Title/Abstract] OR Virtual game*[Title/Abstract] OR Virtual reality game*[Title/Abstract] OR VR game*[Title/Abstract] OR Augmented reality game*[Title/Abstract] OR “AR game*”[Title/Abstract] OR Mobile game*[Title/Abstract] OR Television game*[Title/Abstract] OR TV game*) AND ((Cognitive Dysfunction [MeSH Terms] OR Cognit* [Title/Abstract] OR Executive function*[Title/Abstract] OR Memory defect*[Title/Abstract] OR Attention defect*[Title/Abstract] OR Visual spatial [Title/Abstract] OR Reasoning skill [Title/Abstract] OR Problem solv*[Title/Abstract] OR Process* speed [Title/Abstract] OR Spatial navigation [Title/Abstract]) AND (Rehabilitation [MeSH Terms] OR Therapeutics [MeSH Terms] OR Remediat*[Title/Abstract] OR Rehabilitat*[Title/Abstract] OR Train*[Title/Abstract] OR Therap*[Title/Abstract] OR Readapt*[Title/Abstract]) OR (Cognitive Remediation [MeSH Terms] OR Cognitive Behavioral Therapy [MeSH Terms])) |
| 1. **Scopus search strategy:** |
| (TITLE-ABS-KEY (computer pre/1 game*) OR TITLE-ABS-KEY (video pre/1 game*) OR TITLE-ABS-KEY (videogame*) OR TITLE-ABS-KEY (online game*) OR TITLE-ABS-KEY (applied game*) OR TITLE-ABS-KEY (serious game*) OR TITLE-ABS-KEY (gamification) OR TITLE-ABS-KEY (simulation game*) OR TITLE-ABS-KEY (Virtual pre/1 game*) OR TITLE-ABS-KEY (VR game*) OR TITLE-ABS-KEY (Augmented reality game*) OR TITLE-ABS-KEY (AR game*) OR TITLE-ABS-KEY (Mobile game*) OR TITLE-ABS-KEY (Television game*) OR TITLE-ABS-KEY (TV game*)) AND ((TITLE-ABS-KEY (Cognit*) OR TITLE-ABS-KEY (Executive function*) OR TITLE-ABS-KEY (Memory defect*) OR TITLE-ABS-KEY (attention defect*) OR TITLE-ABS-KEY (visual spatial) OR TITLE-ABS-KEY (Reasoning skill) OR TITLE-ABS-KEY (Problem solv*) OR TITLE-ABS-KEY (Process* speed) OR TITLE-ABS-KEY (Spatial navigation)) AND (TITLE-ABS-KEY (Rehabilitat*) OR TITLE-ABS-KEY (therap*) OR TITLE-ABS-KEY (Remediat*) OR TITLE-ABS-KEY (train*) OR TITLE-ABS-KEY (Readapt*))) |
| 1. **Embase search strategy:** |
| (‘videogame’/de OR ‘computer NEAR/1 game*’:ab,ti OR ‘video NEAR /1 game*’:ab,ti OR ‘videogame*’:ab,ti OR ‘online game*’:ab,ti OR ‘applied game*’:ab,ti OR ‘serious game*’:ab,ti OR ‘gamification’:ab,ti OR ‘simulation game*’:ab,ti OR ‘Virtual near/1 game*’:ab,ti OR ‘Virtual reality game*’:ab,ti OR ‘VR game*’:ab,ti OR ‘Augmented reality game*’:ab,ti OR ‘AR game*’:ab,ti OR ‘Mobile game*’:ab,ti OR ‘Television game*’:ab,ti OR ‘TV game*’:ab,ti) AND (((‘Cognit*’:ab,ti OR ‘Executive function*’:ab,ti OR ‘memory defect*’:ab,ti OR ‘attention defect*’:ab,ti OR ‘visual spatial’:ab,ti OR ‘Reasoning skill’:ab,ti OR ‘problem solv*’:ab,ti OR ‘Process* speed’:ab,ti OR ‘spatial navigation’:ab,ti ) AND (‘Remediat*’:ab,ti OR ‘Rehabilitat*’:ab,ti OR ‘Train*’:ab,ti OR ‘Therap*’:ab,ti OR ‘readapt*’:ab,ti)) OR (‘Cognitive Remediation Therapy’/de OR ‘Cognitive Rehabilitation’/de OR ‘Cognitive Therapy’/de OR ‘Neurorehabilitation’/de)) |
| 1. **ISI search strategy:** |
| TS= (computer NEAR/1 game* OR video NEAR/1 game* OR videogame* OR online game* OR applied game* OR serious game* OR gamification OR simulation game* OR Virtual NEAR/1 game* OR VR game* OR Augmented reality game* OR AR game* OR Mobile game* OR Television game* OR TV game*) AND (TS= (Cognit* OR Executive function* OR Memory defect* OR attention defect* OR Visual spatial OR reasoning skill OR problem solv* OR process* speed OR spatial navigation*) AND TS= (Remediat* OR Rehabilitation* OR Train* OR Therap* OR Readapt*)) |
| 1. **IEEE search strategy:** |
| ("Full Text Only":video game) AND ("Full Text Only":cognitive rehabilitation) |
| 1. **Cochrane search strategy:** |
| "serious game*" OR "gamification" OR "simulation game" OR "Virtual game" OR "VR game" OR "Augmented reality game" OR "video game*" AND "Cognit*" OR "Executive function" OR "Memory defect" OR "attention defect" OR "Visual spatial" OR "reasoning skill" OR "problem solv*" OR "process* speed" OR "spatial navigation*" AND "Remediat*" OR "Rehabilitation*" OR "Train*" OR "Therap*" OR "Readapt*" |

Table S3- Steps to search different databases and their results

| **Num** | **Steps** | **Included paper in each step** | | | | | | |
| --- | --- | --- | --- | --- | --- | --- | --- | --- |
|  |  | **Scientific Databases** | | | | | | **Total** |
|  |  | **PubMed** | **Scopus** | **Embase** | **ISI** | **IEEE** | **Cochrane** |  |
| 1 | Primary search | 975 | 1682 | 841 | 1617 | 1394 | 406 | 6915 |
| 2 | Only journal article | 257 | 1664 | 527 | 1372 | 286 | 406 | 4512 |
| 3 | English language | 251 | 1549 | 515 | 1294 | - | 406 | 4015 |
| 4 | Date Range (2011-2021) | 223 | 1362 | 511 | 1188 | 247 | 406 | 3937 |
| 5 | Remove duplications | 29 | 541 | 122 | 506 | 234 | 240 | 1672 |
| 6 | Screening of title & abstract | 9 | 83 | 8 | 85 | 9 | 109 | 303 |
| 7 | Full text downloading | 9 | 81 | 7 | 85 | 9 | 53 | 244 |
| 8 | Screening of Fulltext | 7 | 49 | 5 | 42 | 3 | 18 | 124 |
| 9 | Extraction Data | 3 | 6 | 2 | 6 | 1 | 3 | **21** |
| **Num** | **Steps** | **Hand Searching** | | | | | | **Total** |
|  |  | **References of articles** | | | **Scholar google** | | |  |
| 1 | Primary search | 40 | | | 51 | | | 91 |
| 2 | Screening of title & abstract | 36 | | | 37 | | | 73 |
| 3 | Full text downloading | 19 | | | 30 | | | 49 |
| 4 | Screening of Full text | 18 | | | 26 | | | 44 |
| 5 | Extraction Data | 3 | | | 6 | | | **9** |
